# Current Status of Outcomes Reported by Patients With Stroke and an Analysis of Influencing Factors: Cross-Sectional Questionnaire Study

**DOI:** 10.2196/58330

**Published:** 2024-08-26

**Authors:** Jia Sun, Liang Ma, Xiao Miao, Hui Sun, SuSu Zhu, Ran Zhang, LeLe Fan, TingTing Hu

**Affiliations:** 1 Nursing Department The Affiliated Lianyungang Hospital of Xuzhou Medical University Lianyungang China; 2 Department of Medicine Southeast University Nanjing China

**Keywords:** stroke, patient-reported outcomes, blood lipids, influence factor, correlation analysis, nursing care

## Abstract

**Background:**

Stroke is the leading cause of acquired disability and the second leading cause of death worldwide. Its rate of incidence, disability, mortality, and recurrence is high, and the patients experience various symptoms of discomfort, which not only affect their rehabilitation function but also reduce their ability to perform daily activities and their quality of life. Nowadays, with the improvement of China’s medical standards, patients are increasingly attentive to their quality of life and health status. However, diagnostic techniques and effective treatments for patients with stroke are still limited but urgently required.

**Objective:**

This study aimed to evaluate the quality of life during hospitalization using a stroke patient-reported outcomes (PROs) scale and additionally to recognize potential factors and risk indicators that may impact recurrent events, facilitating early intervention measures.

**Methods:**

This is a registry-based, retrospective observational cross-sectional study on patients with stroke. A convenient sampling method was used to select various indicators of patients. The Stroke-PRO scale was then used to assess patients’ conditions across physical, psychological, social, and therapeutic domains. Multiple linear regression analysis was applied to identify factors influencing stroke PROs, while correlation analysis was conducted to explore the relationship between these outcomes and blood lipid levels.

**Results:**

The mean Stroke-PRO score in this study was 4.09 (SD 0.29) points. By multiple linear regression analysis, residence, occupation, physical exercise, Barthel index, Braden scale, National Institutes of Health Stroke Scale scores at admission, and stroke type were the risk factors for reported outcomes of patients with stroke (*P*<.05). Correlation analysis showed that serum triglyceride, total cholesterol, and low-density lipoprotein were negatively correlated with Stroke-PRO scores in patients with stroke (*P*<.05), while high-density lipoprotein was positively correlated with patients with stroke (*P*<.05). The 95% CI was –0.31 to –0.03 for triglyceride, 0.17-0.44 for high-density lipoprotein, –0.29 to –0.01 for cholesterol, –0.30 to –0.02 for low-density lipoprotein, and –0.12 to 0.16 for blood glucose.

**Conclusions:**

Patients with stroke have a low level of health, and their reported outcomes need to be improved. Accordingly, nursing staff should pay attention to the quality of life and blood lipid indexes of patients with stroke, actively assess their actual health status, and take early intervention measures to promote their recovery.

## Introduction

Cerebral stroke, also known as “stroke” or cerebrovascular accident, is an acute cerebrovascular disease caused by various factors, leading to the rupture or blockage of cerebral blood vessels. It primarily results in ischemic or hemorrhagic damage to brain tissue [1]. Stroke can be categorized into 3 distinct subtypes, 87% of which are ischemic stroke (IS), followed by 10% hemorrhagic stroke (HS) and 3% subarachnoid stroke [2]. Stroke poses a severe threat to human health, ranking as the leading cause of acquired disability and adult mortality in China [3], and the second leading cause of death worldwide [4,5]. About 50% of patients with stroke have reduced mobility, and 26% remain disabled in basic activities of daily living after a stroke [6]. With increasing recurrence rates, stroke is characterized by a high incidence, high disability rate, high mortality rate, high recurrence rate, and substantial economic burden [7]. Using data from the Global Burden of Disease Study conducted in 2021, Ma et al [8], revealed a decline of 9.3% (95% CI 3.3-15.5) in age-standardized incidence and 39.8% (95% CI 28.6-50.7) in mortality rates from 1990 to 2019.

Patients experience various symptoms, including physical and psychological manifestations, during medical disease treatment and rehabilitation [9-11]. These symptoms not only impact the recovery functions of the patients but also reduce their ability to perform daily activities and quality of life, imposing a heavy burden on family caregivers and society at large. Until now, there is no effective treatment for stroke, and prevention is the most feasible strategy to reduce the harm of stroke and reduce its social burden. As we know, the 5 leading risk factors for stroke are high systolic blood pressure, high BMI, high fasting plasma glucose, ambient particulate matter pollution, and smoking [12-14]. In addition, drinking, aging population, physical inactivity, and metabolic problems also affect the incidence of stroke [15]. According to the World Health Organization, effective stroke prevention strategies include targeting modifiable risk factors, such as hypertension, control of elevated lipids, diabetes, smoking, low physical activity, unhealthy diet, and abdominal obesity [6]. Therefore, early identification of risk factors is essential to prevent stroke.

Foreign studies on patient-reported outcomes (PROs) originated in the 1970s. The US Food and Drug Administration defines PROs as “all information about a patient’s health status that comes directly from the patient and does not require interpretation by a doctor or others” [16,17]. Often in the form of self-assessment scales, PROs comprehensively assess outcomes, such as symptom burden, emotional status, functional level, and quality of life related to the disease and treatment, offering a holistic understanding of patients’ health levels [18,19]. Accordingly, PROs are usually more sensitive than methods such as physician interviews or scoring. Considering limited research in China, the applicability of PROs in patients with stroke still requires further validation.

Although the qualitative research findings are from our single institution, this study is of vital significance for the accurate identification and effective management of symptoms of patients with stroke using comprehensive assessment methods. Therefore, this study aims to use patient self-assessment reports on health-related outcomes to identify changes in the status of patients with stroke early and improve disease progression, which could be broadly distributed to a diverse sample of stroke caregivers.

## Methods

### Study Design and Sample

Convenience sampling was used to select patients with stroke from the Neurology and Neurosurgery Department of a tertiary hospital between May 2023 and September 2023 in Lianyungang City, Jiangsu Province, China. Details of participant inclusion and exclusion criteria are shown in Textbox 1.

**Participant inclusion and exclusion criteria.**

**Inclusion criteria**

- According to the diagnostic criteria for stroke outlined in the “Diagnosis of various major cerebrovascular disease in China 2019” [20]. The patients were confirmed through head computed tomography or (and) magnetic resonance imaging examinations
- Age >40 years
- Clear consciousness, without cognitive or communication impairments
- Informed consent and voluntary participation in the study
- Complete clinical data

**Exclusion criteria**

- Patients who are unconscious, have language or intellectual impairments preventing comprehension or questionnaire completion, or refuse to participate in this project are excluded

### Procedure and Data Collection

Based on multivariate linear regression analysis, the sample size was set at 5-10 times the number of independent variables [21]. Considering 16 influence factors and a 15% (15/100) expected loss rate in this study, the calculated sample size ranged from 96 to 195 cases.

### Demographic and Clinical Variables

Questionnaires were used to collect demographic information, including age, sex, body mass index, occupation, marital status, monthly family income, level of education, residence, smoking history, and alcohol consumption history. Furthermore, we placed a specific focus on collecting relevant patient medical history, including a history of hypertension, diabetes, heart disease, and stroke. Simultaneously, laboratory data, such as fasting blood glucose, triglycerides, high-density lipoprotein cholesterol, low-density lipoprotein cholesterol (LDL-C), and total cholesterol, were also collected to gain insights into the condition during the hospitalization of the patients.

### Stroke Patient-Reported Outcomes

The Stroke-PRO scale, developed by Professor Yan Bo Zhang from Shanxi Medical University, was used to gather symptom data [22]. The scale is divided into 46 items consisting of 10 dimensions across 4 domains, with Likert 5-point scale ratings. Responses are scaled from 0 to 4 points, using a unique calculation process for positive and negative items. Positive item scores are calculated as 1 plus the actual score, and negative item scores are calculated as 5 minus the actual score. Each item has a score range of 1-5 points. As the number of items varies across dimensions and domains, direct comparison of 5-point scores within different dimensions and domains is not possible. Therefore, the scale score is computed by summing all item scores and dividing by the total number of items on the scale. The total score reflects the patient’s overall quality of life, with higher scores indicating better quality of life and lower scores indicating diminished quality. Previous research has demonstrated the scale’s strong reliability and validity, with Cronbach coefficients for the 4 domains (physiological, psychological, social, and therapeutic) being 0.888, 0.908, 0.879, and 0.861, respectively, implying high reliability [23].

### Data Collection and Quality Control Methods

Our research team defined the inclusion and exclusion criteria and 2 trained investigators explained the unified guidance to the patients who meet the standards. The investigators responsible for patient screening and recruitment underwent training to strictly adhere to clear inclusion and exclusion criteria for subjects. Detailed documentation of each patient’s screening process, including reasons for inclusion and exclusion, was meticulously maintained to minimize bias. After seeking informed consent from patients, questionnaires were distributed on site and patients were asked to fill them out independently. During this, the investigators only explain what the patient does not understand. The general data of the patients were obtained from the medical records by the investigators, and the clinical objective indicators were collected from the hospital’s electronic medical record system of the patients, along with the social demographic data, disease, and treatment-related data.

### Statistical Analysis

Data analysis was conducted using SPSS 27.0 (IBM Corp) statistical software. Normality tests were applied for continuous data, with normally distributed continuous data described as mean and SD, and nonnormally distributed data presented as median with the 25th and 75th percentile. Descriptive statistics, including frequency and percentage, were used for categorical data. Single-factor analysis of variance and multiple linear regression were used to analyze the influence factors of PROs in patients with stroke. Pearson correlation analysis was performed to explore the correlation between PROs and lipid profiles. A significance level of *P*<.05 is considered statistically significant.

### Ethical Considerations

In this study, all the methods used by the researcher throughout the questionnaire study were carried out in accordance with the relevant guidelines and regulations. This study was approved by the Clinical Research Ethics Committee of the First People’s Hospital of Lianyungang (approval number KY-20230215002-01). Furthermore, it can be demonstrated that the research was conducted in accordance with the standards set out in the 1964 Declaration of Helsinki. All participants in this study were young and middle-aged with normal consciousness and cognitive abilities, and all participants signed an informed consent form.

## Results

### Status of Patient-Reported Outcomes in Patients With Stroke

A total of 195 patients were included in this study, comprising 123 (63.1%) male patients and 72 (36.9%) female patients, with an average age of 66.77 (SD 10.82) years. The mean Stroke-PRO scale score for patients with stroke was 4.09 (SD 0.29) points. Specifically, the mean scores for the physiological domain, psychological domain, social domain, and therapeutic domain were 18.57 (SD 2.22), 15.78 (SD 0.9), 6.46 (SD 0.55), and 5.22 (SD 0.39) points, respectively.

### Single-Factor Analysis of Patient-Reported Outcomes in Patients With Stroke

Among patients with stroke, there were statistical differences (*P*<.05) in Stroke-PRO scores with different residential areas, occupations, physical exercise habits, stroke types, Barthel index, Braden scale, and National Institutes of Health Stroke Scale (NIHSS) scores at admission (Table 1).

**Table 1:** Single-factor analysis of general information and patient-reported outcomes in patients with stroke (N=195).

Variables		Cases, n (%)	PRO^a^ score (point), mean (SD)	*t* test *(df)*	*P* value
**Gender**				1.393 (194)	.24
	Men	123 (63.1)	4.11 (0.29)		
	Women	72 (36.9)	4.06 (0.28)		
**Age (years)**				1.002 (194)	.48
	＜50	9 (4.6)	4.11 (0.25)		
	50-69	104 (53.3)	4.14 (0.28)		
	＞70	82 (42.1)	4.02 (0.28)		
**BMI (kg/m^2^)**				1.015 (194)	.47
	＜18.5	5 (2.6)	3.84 (0.29)		
	18.5-23.9	56 (28.7)	4.08 (0.28)		
	24-27.9	95 (48.7)	4.09 (0.29)		
	≥28	39 (20.0)	4.12 (0.28)		
**Educational level**				1.967 (194)	.12
	Elementary school	77 (39.5)	4.07 (0.28)		
	Junior high school	74 (38.0)	4.05 (0.28)		
	Technical or high school	30 (15.4)	4.18 (0.3)		
	College and above	14 (7.2)	4.17 (0.32)		
**Residence**				6.427 (194)	*.01* ^b^
	Rural	82 (42.1)	4.03 (0.28)		
	Town	113 (57.9)	4.13 (0.29)		
**Smoking history**				0.940 (194)	.39
	Yes	66 (33.8)	4.07 (0.27)		
	No	112 (57.4)	4.11 (0.28)		
	Former smoker	17 (8.7)	4.02 (0.39)		
**Alcohol history**				1.775 (194)	.17
	Yes	72 (36.9)	4.13 (0.29)		
	No	112 (57.4)	4.07 (0.26)		
**Occupation**				3.678 (194)	*.01*
	Former drinker	11 (5.6)	3.98 (0.44)		
	Unemployed	21 (10.7)	4.02 (0.27)		
	Retired	77 (39.4)	4.12 (0.29)		
	Employed	38 (19.5)	4.19 (0.27)		
	Farmer	59 (30.2)	4.02 (0.28)		
**Physical exercise**				7.522 (194)	*＜* *.001*
	None	98 (50.2)	4.02 (0.28)		
	Frequent	42 (21.5)	4.2 (0.31)		
	Occasional	55 (28.2)	4.14 (0.25)		
**Polypharmacy**				2.447 (194)	.12
	Yes	131 (67.1)	4.11 (0.3)		
	No	64 (32.8)	4.04 (0.25)		
**Stroke type**				3.306 (194)	*.04*
	Subarachnoid hemorrhage	16 (8.2)	4.04 (0.24)		
	Intracerebral hemorrhage	41 (21.0)	4 (0.2)		
	Ischemic stroke	138 (70.7)	4.12 (0.31)		
**High blood pressure**				0.899 (194)	.34
	Yes	151 (77.4)	4.1 (0.29)		
	No	44 (22.6)	4.05 (0.26)		
**Diabetes**				1.584 (194)	.21
	Yes	59 (30.3)	4.13 (0.3)		
	No	136 (69.7)	4.07 (0.28)		
**Heart disease**				1.969 (194)	.16
	Yes	26 (13.3)	4.02 (0.27)		
	No	169 (86.7)	4.1 (0.29)		
**Duration of complications (years)**				1.069 (194)	.38
	≤1	34 (17.4)	4.09 (0.24)		
	2-5	36 (18.5)	4.07 (0.24)		
	6-9	14 (7.2)	4.11 (0.32)		
	≥10	111 (56.9)	4.1 (0.31)		
**Stroke history**				1.224 (194)	.27
	Yes	119 (61.1)	4.11 (0.26)		
	No	76 (38.9)	4.06 (0.32)		
**NIHSS^c^ (point)**				6.236 (194)	*＜* *.001*
	0: normal	53 (27.2)	4.32 (0.23)		
	1-4: minor apoplexy	73 (37.4)	4.09 (0.26)		
	5-15: moderate apoplexy	51 (26.2)	3.9 (0.24)		
	16-20: moderate-to-vigorous apoplexy	9 (4.6)	3.93 (0.2)		
	21-42: vigorous apoplexy	9 (4.6)	3.94 (0.14)		
**B** **arthel index**				8.196 (194)	*＜* *.001*
	0-40	90 (46.2)	3.92 (0.22)		
	41-60	26 (13.3)	4.05 (0.29)		
	61-99	75 (38.5)	4.29 (0.22)		
	100	4 (2.0)	4.35 (0.23)		
**Braden scale**				11.365 (194)	*＜* *.001*
	10-12	11 (5.6)	3.79 (0.16)		
	13-14	20 (10.3)	3.84 (0.23)		
	15-18	45 (23.1)	3.96 (0.27)		
	19-23	119 (61.0)	4.21 (0.24)		

^a^PRO: patient-reported outcome.

^b^The bold values indicate statistical significance with a *P*<.05.

^c^NIHSS: National Institutes of Health Stroke Scale.

### Multivariate Analysis of Patient-Reported Outcomes in Patients With Stroke

Using the Stroke-PRO score as the dependent variable, a multiple regression analysis was conducted with variables that showed statistical significance in the single-factor analysis as independent variables [24]. The assignment of values for independent variables is shown in Table 2. The results revealed that Barthel index, Braden scale, and NIHSS scores at admission were significant factors influencing PROs in patients with stroke (*P*<.05; Table 3).

**Table 2:** Assignment of argument variables.

Independent variables	Assignment
Residence	Town=1, rural=2
Occupation	Employed=1, retired=2, farmer=3, unemployed=4
Physical exercise	Frequent=1, none=2, occasional=3
Stroke type	Subarachnoid hemorrhage=1, intracerebral hemorrhage=2, ischemic stroke=3

**Table 3:** Multiple linear regression analysis of reported outcomes of patients with stroke (N=195).

Model	β	SE	β	*t* test (*df*)	*P* values	95% CI
Constant	1.139	0.042	—^a^	27.168 (194)	<.001	—
Barthel	0.002	0.000	0.420	4.754 (194)	＜.001	0.30-3.27
Braden	0.009	0.003	0.287	3.389 (194)	＜.001	0.33-3.0
NIHSS^b^	–0.002	0.001	–0.105	–1.780 (194)	.08	0.69-1.46

^a^Not applicable.

^b^NIHSS: National Institutes of Health Stroke Scale.

### Correlation Analysis Between Patient-Reported Outcomes and Lipid Profiles in Patients With Stroke

The correlation analysis revealed a negative correlation between triglycerides, cholesterol, low-density lipoprotein, and Stroke-PRO scores in patients with stroke (all *P*<.05). Conversely, there was a positive correlation between the high-density lipoprotein and Stroke-PRO scores (*P*<.05; Table 4).

**Table 4:** Correlation between patient-reported outcomes and blood lipid indexes in patients with stroke (N=195)

Blood lipid indexes	Pearson *r*	95% CI	*P* value
Triglyceride	–0.172^**^	–0.31 to –0.03	.02
High-density lipoprotein	0.304^*^	0.17 to 0.44	＜.001
Cholesterol	–0.148^**^	–0.29 to –0.01	.04
Low-density lipoprotein	–0.157^**^	–0.30 to –0.02	.03
Fasting blood glucose.	0.020	–0.12 to 0.16	.79

^*^P.05; ^**^P<.001.

## Discussion

### Principal Findings

The study reveals PRO scores across various domains, which are physiological (mean 18.57, SD 2.22), psychological (mean 15.78, SD 0.9), social (mean 6.46, SD 0.55), and treatment (mean 5.22, SD 0.39). However, the diverse nature of stroke cases complicates direct score comparison across domains despite using the Likert 5-point scoring system. Generally, patients with stroke face a lower quality of life, likely due to the prolonged disease course, multiple complications, substantial family burdens, and physical functional impairments [25]. In addition, domestic research has indicated that a high score means more severe symptoms and therefore a poorer quality of life in patients with stroke [26]. The severity of stroke within 3-5 days after admission is an independent risk factor for poor quality of life among survivors (*P*<.001). Therefore, it is an effective approach to enhancing the quality of life for patients with IS by improving symptoms as soon as possible [27]. Although there is existing domestic and international research on PROs, standardized measurement tools for assessing outcomes in patients with stroke have not been established.

The study underscores the impact of residence on the outcome reports of patients with stroke, revealing higher Stroke-PRO scores in urban residents compared with rural counterparts. This disparity may arise from varying health awareness, cultural literacy, and medical conditions in rural areas. In line with the findings by Tu et al [28], higher stroke rates in rural areas are linked to an imbalanced distribution of risk factors compared with urban areas. Rural participants exhibit higher proportions of individuals with lower education and monthly income levels, as well as a lower prevalence of hypertension, diabetes, and abnormal lipid levels. Conversely, they possess higher rates of smoking, alcohol consumption, lack of physical activity, and obesity. Rural areas also exhibit lower awareness, treatment, and control rates for hypertension, diabetes, and abnormal lipid levels than urban areas. Therefore, health care providers should prioritize health education, disseminate relevant knowledge, and enhance patient consciousness to improve quality of life.

Unemployed patients report lower health levels and quality of life, suggesting that unemployment significantly impacts health outcomes. The difference may be related to several factors, such as job nature, psychological stress, economic income, and life pressures. Accordingly, special attention to the psychological and economic conditions of patients, coupled with patient-centric approaches, such as active listening and counseling, is crucial for encouraging self-management and fostering a positive mindset.

Physical exercise significantly influences the outcomes of patients with stroke, and those lacking exercise exhibit lower Stroke-PRO scores, indicating poorer self-reported health and quality of life. Accumulating evidence has demonstrated that exercise can improve risk factors associated with initial or recurrent stroke, and good exercise has a positive effect on neuroplasticity, thus potentially improving cognition and function. Especially, moderate- to high-intensity exercise after a stroke can reduce the risk of stroke recurrence and improve depression of the patients [28-30]. Collectively, health care providers should stress the importance of functional exercise, creating personalized plans to enhance physical fitness and prevent strokes.

The widely used Barthel index assesses activities of daily living, determining recovery needs and care levels. The Braden score gauges pressure ulcer risk, requiring increased attention to patients’ ability to perform daily activities. Studies indicate the initial NIHSS score influences outcomes, aligning with clinical experience. During nursing, emphasis on the NIHSS score and personalized care plans aids patient recovery.

Furthermore, the study establishes a link between abnormal blood lipids and stroke occurrence. Dyslipidemia is a major atherosclerotic cardiovascular disease risk factor linked to stroke incidence [31]. Mechanisms triggering atherosclerosis, especially hypercholesterolemia, contribute to coronary heart disease. LDL-C is the end product of lipoprotein metabolism, and a higher plasma level of LDL-C is a major risk factor for atherosclerotic cardiovascular disease [32]. Elevated total cholesterol levels increase ischemic cardiovascular disease risk. Conversely, high-density lipoprotein, an antiatherosclerotic lipid, correlates negatively with stroke severity and prognosis, and LDL-C levels positively correlate with IS risk but negatively with HS [33]. Similarly, high-density lipoprotein cholesterol levels negatively correlate with IS but not hemorrhagic stroke, while triglyceride levels show a weak positive correlation with IS and a negative correlation with HS [34].

Considering the above, this study focuses on the relationship between Stroke-PRO scores in patients with stroke and blood lipid indicators. Results indicate a negative correlation of triglycerides, cholesterol, and LDL with Stroke-PRO scores, but a positive correlation of high-density lipoprotein with Stroke-PRO scores. This conclusion aligns with the results of the aforementioned study. Therefore, for individuals at high risk of cerebrovascular risk factors, it is recommended that patients reduce stroke risk through smoking cessation, alcohol moderation, regular exercise, a balanced diet, routine follow-ups, weight control, and stable lipid and blood pressure levels [35].

### Limitations

First, compared with commonly used quality-of-life scales, the usage of the Stroke-PRO scale is relatively limited, suggesting a need for broader applicability. Second, the Stroke-PRO scale was initially developed within a specific linguistic and cultural context in China. When used in different linguistic and cultural settings, even after translation and cultural adaptation, there may still be comprehension biases. Finally, patients’ health status may change over time, as Stroke-PRO assessments are typically conducted at specific time points. Thus, this approach may not capture the dynamic changes in the health of the patients throughout the entire rehabilitation process.

While our study used a convenient sampling method, potentially introducing bias, we mitigated this limitation by furnishing detailed descriptions of key sample characteristics—age, gender, and clinical condition—and comparing them to population norms for the assessment of the readers. Notably, future research should prioritize larger sample sizes for greater representativeness, facilitating early intervention and dynamic tracking of health outcomes in patients with stroke.

### Conclusion

Given the observed lower health levels, the study underscores the imperative to enhance PROs in stroke care. Significant correlations of characteristics of patients with health levels emphasize the crucial role of lipid management in stroke care. Moreover, accurate patient reporting emerges as a key contributor to improved functional outcomes, enhanced quality of life, and reduced correlation with risk factors.

## Abbreviations

HS: hemorrhagic stroke
IS: ischemic stroke
LDL-C: low-density lipoprotein cholesterol
NIHSS: National Institute of Health Stroke Scale
PRO: patient-reported outcome

## Data Availability

The data used in this study were collected through a questionnaire designed and conducted by the authors themselves. All data generated or analyzed during this study are included in this published article.

## References

[1] Hailan LX, Wang F (2023). Research progress on disease cognition in stroke patients. Chin Evidence Based Nurs.

[2] Lackland DT, Roccella EJ, Deutsch AF, Fornage M, George MG, Howard G, Kissela BM, Kittner SJ, Lichtman JH, Lisabeth LD, Schwamm LH, Smith EE, Towfighi A (2014). Factors influencing the decline in stroke mortality: a statement from the American Heart Association/American Stroke Association. Stroke.

[3] Group Rospti CW (2020). Brief report on stroke prevention and treatment in China 2019. Chin J Cerebrovascular Dis.

[4] GBD 2019 Stroke Collaborators (2021). Global, regional, and national burden of stroke and its risk factors, 1990-2019: a systematic analysis for the global burden of disease study 2019. Lancet Neurol.

[5] Owolabi MO, Thrift AG, Martins S, Johnson W, Pandian J, Abd-Allah F, Varghese C, Mahal A, Yaria J, Phan HT, Roth G, Gall SL, Beare R, Phan TG, Mikulik R, Norrving B, Feigin VL, Stroke Experts Collaboration Group (2021). The state of stroke services across the globe: report of world stroke organization-world health organization surveys. Int J Stroke.

[6] Katan M, Luft A (2018). Global burden of stroke. Semin Neurol.

[7] ROS Prevention, TiCW Group (2023). Brief report on stroke prevention and treatment in China 2020. Chin J Cerebrovascular Dis.

[8] Ma Q, Li R, Wang L, Yin P, Wang Y, Yan C, Ren Y, Qian Z, Vaughn MG, McMillin SE, Hay SI, Naghavi M, Cai M, Wang C, Zhang Z, Zhou M, Lin H, Yang Y (2021). Temporal trend and attributable risk factors of stroke burden in China, 1990-2019: an analysis for the global burden of disease study 2019. Lancet Public Health.

[9] Dou DM, Huang LL, Dou J, Wang XX, Wang PX (2018). Post-stroke depression as a predictor of caregivers burden of acute ischemic stroke patients in China. Psychol Health Med.

[10] McCrory M, Murphy DF, Morris RC, Noad RF (2023). Evaluating the GAD-2 to screen for post-stroke anxiety on an acute stroke unit. Neuropsychol Rehabil.

[11] Turner GM, McMullan C, Atkins L, Foy R, Mant J, Calvert M (2019). TIA and minor stroke: a qualitative study of long-term impact and experiences of follow-up care. BMC Fam Pract.

[12] Feigin VL, Brainin M, Norrving B, Martins S, Sacco RL, Hacke W, Fisher M, Pandian J, Lindsay P (2022). World stroke organization (WSO): global stroke fact sheet 2022. Int J Stroke.

[13] Wang L, Zhou B, Zhao Z, Yang L, Zhang M, Jiang Y, Li Y, Zhou M, Wang L, Huang Z, Zhang X, Zhao L, Yu D, Li C, Ezzati M, Chen Z, Wu J, Ding G, Li X (2021). Body-mass index and obesity in urban and rural China: findings from consecutive nationally representative surveys during 2004-18. Lancet.

[14] Wang Z, Hu S, Sang S, Luo L, Yu C (2017). Age-period-cohort analysis of stroke mortality in China: data from the global burden of disease study 2013. Stroke.

[15] Wang Y, Li Z, Gu H, Zhai Y, Jiang Y, Zhao X, Wang Y, Yang X, Wang C, Meng X, Li H, Liu L, Jing J, Wu J, Xu A, Dong Q, Wang D, Zhao J, China Stroke Statistics 2019 Writing Committee (2020). China stroke statistics 2019: a report from the national center for healthcare quality management in neurological diseases, China national clinical research center for neurological diseases, the Chinese stroke association, national center for chronic and non-communicable disease control and prevention, Chinese center for disease control and prevention and institute for global neuroscience and stroke collaborations. Stroke Vasc Neurol.

[16] U.S. Department of Health Human Services FDA Center for Drug Evaluation Research, U.S. Department of Health Human Services FDA Center for Biologics Evaluation Research, U.S. Department of Health Human Services FDA Center for Devices Radiological Health (2006). Guidance for industry: patient-reported outcome measures: use in medical product development to support labeling claims: draft guidance. Health Qual Life Outcomes.

[17] Nelson EC, Eftimovska E, Lind C, Hager A, Wasson JH, Lindblad S (2015). Patient reported outcome measures in practice. BMJ.

[18] Michniacki TF, Merz LE, McCaffery H, Connelly JA, Walkovich K (2021). Quality of life and patient-reported outcomes in chronic severe neutropenia conditions. Int J Hematol.

[19] Phillips F, Prezio E, Miljanic M, Henneghan A, Currin-McCulloch J, Jones B, Kvale E, Goodgame B, Eckhardt SG (2022). Patient reported outcomes affecting quality of life in socioeconomically disadvantaged cancer patients. J Psychosoc Oncol.

[20] Neurology CSo, Society CS (2019). Diagnostic criteria of cerebrovascular diseases in China (version 2019). Chinese Journal of Neurology.

[21] Ni PC (2010). Liu Na. Sample Size Estimation for Quantitative Research in Nursing Research Ni Ping, Chen Jingli, Liu Na.

[22] Yang J (2013). Development and Evaluation of Patient-Reported Outcome Measures for Stroke Patients.

[23] Yanhong WXYJL (2015). Evaluation of patient-reported outcomes scale for stroke. Xuzhou Medical University.

[24] Jinlian CMCMNYQ (2022). The current status of patient reported outcomes and its influencing factors in patients with stable chronic obstructive pulmonary disease. Chin J Nurs.

[25] Amtmann D, Bamer AM, Kim J, Chung H, Salem R (2018). People with multiple sclerosis report significantly worse symptoms and health related quality of life than the US general population as measured by PROMIS and NeuroQoL outcome measures. Disabil Health J.

[26] Kamwesiga JT, von Koch L, Kottorp A, Guidetti S (2016). Cultural adaptation and validation of stroke impact scale 3.0 version in Uganda: a small-scale study. SAGE Open Med.

[27] Bangzhong SDLZYJL (2019). Relationship research on symptom burden and quality of life elderly stroke patients. Chin Nursing Res.

[28] Tu WJ, Zhao Z, Yin P, Cao L, Zeng J, Chen H, Fan D, Fang Q, Gao P, Gu Y, Tan G, Han J, He L, Hu B, Hua Y, Kang D, Li H, Liu J, Liu Y, Lou M, Luo B, Pan S, Peng B, Ren L, Wang L, Wu J, Xu Y, Xu Y, Yang Y, Zhang M, Zhang S, Zhu L, Zhu Y, Li Z, Chu L, An X, Wang L, Yin M, Li M, Yin L, Yan W, Li C, Tang J, Zhou M, Wang L (2023). Estimated burden of stroke in China in 2020. JAMA Netw Open.

[29] Aguiar LT, Nadeau S, Britto RR, Teixeira-Salmela LF, Martins JC, Samora GAR, da Silva Júnior JA, Faria CDCDM (2020). Effects of aerobic training on physical activity in people with stroke: a randomized controlled trial. NeuroRehabilitation.

[30] Prior PL, Suskin N (2018). Exercise for stroke prevention. Stroke Vasc Neurol.

[31] Jacobson TA, Ito MK, Maki KC, Orringer CE, Bays HE, Jones PH, McKenney JM, Grundy SM, Gill EA, Wild RA, Wilson DP, Brown WV (2015). National lipid association recommendations for patient-centered management of dyslipidemia: part 1—full report. J Clin Lipidol.

[32] Goldstein B, Toth P, Dearborn-Tomazos JL, Giugliano RP, Hirsh BJ, Peña JM, Selim MH, Woo D, American Heart Association Council on Arteriosclerosis‚ Thrombosis Vascular Biology; Council on Cardiovascular Stroke Nursing; Council on Peripheral Vascular Disease; Stroke Council (2023). Aggressive LDL-C lowering and the brain: impact on risk for dementia and hemorrhagic stroke: a scientific statement from the American heart association. Arterioscler Thromb Vasc Biol.

[33] The Writing Committee Of The Report On Cardiovascular Health And Diseases In China (2022). Summary of China cardiovascular health and disease report 2021. Cardio-Cerebrovasc Dis Prev Treat.

[34] Sun L, Clarke R, Bennett D, Guo Y, Walters RG, Hill M, Parish S, Millwood IY, Bian Z, Chen Y, Yu C, Lv J, Collins R, Chen J, Peto R, Li L, Chen Z, China Kadoorie Biobank Collaborative Group, International Steering Committee, International Co-ordinating Centre‚ Oxford, National Co-ordinating Centre‚ Beijing, Regional Co-ordinating Centres (2019). Causal associations of blood lipids with risk of ischemic stroke and intracerebral hemorrhage in Chinese adults. Nat Med.

[35] Kleindorfer DO, Towfighi A, Chaturvedi S, Cockroft KM, Gutierrez J, Lombardi-Hill D, Kamel H, Kernan WN, Kittner SJ, Leira EC, Lennon O, Meschia JF, Nguyen TN, Pollak PM, Santangeli P, Sharrief AZ, Smith SC, Turan TN, Williams LS (2021). 2021 Guideline for the prevention of stroke in patients with stroke and transient ischemic attack: a guideline from the American heart association/American stroke association. Stroke.

